# Deciphering the dynamic gene expression patterns of pollen abortion in a male sterile line of *Avena sativa* through transcriptome analysis at different developmental stages

**DOI:** 10.1186/s12870-021-02881-2

**Published:** 2021-02-18

**Authors:** Lijun Zhang, Mingchuan Ma, Lin Cui, Longlong Liu

**Affiliations:** Crop Germplasms Resources Research Institute, Shanxi Academy of Agricultural Sciences, Taiyuan, China

**Keywords:** Oat, Transcriptome, Male sterile line, Near-isogenic line

## Abstract

**Background:**

Male sterility (MS) has important applications in hybrid seed production, and the abortion of anthers has been observed in many plant species. While most studies have focused on the genetic factors affecting male sterility, the dynamic gene expression patterns of pollen abortion in male sterile lines have not been fully elucidated. In addition, there is still no hybrid oat that is commercially planted due to the lack of a suitable system of male sterility for hybrid breeding.

**Results:**

In this study, we cultivated a male sterile oat line and a near-isogenic line by crossbreeding to elucidate the expression patterns of genes that may be involved in sterility. The first reported CA male sterile (CAMS) oat line was used for cross-testing and hybridization experiments and was confirmed to exhibit a type of nuclear sterility controlled by recessive genes. Oat stamens of two lines were sampled at four different developmental stages separately. Paired-end RNA sequencing was performed for each sample and generated 252.84 Gb sequences. There were 295,462 unigenes annotated in public databases in all samples, and we compared the histological characteristics and transcriptomes of oat stamens from the two oat lines at different developmental stages. Our results demonstrate that the sterility of the male sterile oat line occurs in the early stage of stamen development and is primarily attributable to abnormal meiosis and the excessive accumulation of superoxide.

**Conclusions:**

To the best of our knowledge, this study is the first to decipher the dynamic expression profiles of pollen abortion CAMS and CA male fertile (CAMF) oat lines, which may represent a valuable resource for further studies attempting to understand pollen abortion and anther development in oats.

**Supplementary Information:**

The online version contains supplementary material available at 10.1186/s12870-021-02881-2.

## Background

Commercial breeding based on exploring heterosis has made strong contributions to improvements in fruit size, yield and other quantitative traits. Male sterility is widely utilized in breeding and exhibits considerable advantages in crossbreeding, especially economic advantages. Male sterility in higher plants refers to the inability to produce viable male gametes, which results in the abortion of anthers and sterility in the development of male sex organs. Any changes in pollen development, including a series of physiological and biochemical reactions, may lead to microspore abortion. In plants, male sterility is divided into cytoplasmic male sterility (CMS), genic male sterility (GMS) and genic-cytoplasmic male sterility (GCMS) based on the genetic characteristics [28]. Meanwhile, male sterility is also an important model for studying the development of stamen and pollen and examining cytoplasmic-nuclear genomic interactions [10].

Male sterility has been reported in more than 617 plant species [25, 28], which strongly supports the inheritance pattern of male sterility, and a large number of genes related to male sterility have also been discovered in many plants. It was a milestone when the first CMS line and hybrid combination were cultivated successfully in rice by transferring the male sterility gene from wild rice [54]. Subsequently, a series of CMS lines and GMS lines from different ecological varieties have been constructed [9, 67]. The first wheat CMS line was developed in 1959 H [29], while only limited hybrid wheat was produced by CMS systems, photoperiodic sensitivity sterility systems, and chemical hybridization agents [35, 63]. Compared with maize and rice [35, 65], the commercial crossbreeding of wheat is still restricted by a lack of practical male sterility traits to improve breeding capacities and reduce costs [63].

Oat (*Avena sativa*), which belongs to the tribe Aveneae of the family Gramineae, is an economically important crop for human consumption and animal feed. Oat is also a healthy crop that is beneficial to the human diet, exhibiting such effects as improving gastrointestinal function [55, 64], promoting glucose metabolism [2], and reducing cholesterol [7, 64]. The inflorescences of oat are arranged in loose panicles, comprising a series of flowering branches from the main axis. Oat florets are composed of female ovaries and male stamens, the number of which is a multiple of three, and they are self-fertilized, which results in a low natural hybridization rate for oats. Similar to other species of the family Gramineae, such as wheat and barley, oats bloom from the top to the base of the spike, making it possible to collect stamens of a plant at different developmental stages. Some major efforts have been made to develop CMS and GMS oat lines, such as the cultivated hexaploid oat *A. sativa* [12]. However, to date, no hybrid oat has been commercially planted due to the lack of a suitable system of male sterility for hybrid breeding [27].

To elucidate the molecular mechanism underlying male sterility, in this study, the male sterile oat plant, known as the CA male sterile (CAMS) oat line and controlled by recessive nuclear genes, was employed as a time-course transcriptome strategy. The CAMS oat line and near-isogenic line used in this study were cultivated through long-term breeding for many years. The dynamic gene expression patterns of pollen abortion in the male sterile line are presented, and the highly expressed genes and specific biological processes were further identified by comprehensive transcriptomic analysis. To determine the possible factors responsible for the sterility of the CAMS line, the analysis of networks and associated pathways was combined with experiments on CAMS morphological characteristics, thereby identifying candidate genes involved in pollen abortion and the development of anthers. This work provides strong circumstantial evidence for the effective screening of candidate nuclear recessive gene responses for male fertility in CAMS oat lines and establishes a foundation for large-scale commercial hybrid oat breeding and hybrid seed production.

## Methods

### Plant materials

In this study, sterile oat materials and Stout germplasms were used to generate near-isogenic lines. Plants, stamens, and pollen of the sterile oat materials were photographed in JEOL JSM-35C scanning electron microscope (SEM) operating at 25 kV. Sterile oat plants were first identified in the field by the Shanxi Academy of Agricultural Sciences (Shanxi Province, China) in 1994 and were named the CA male sterile (CAMS) oat line [12]; oat sterility was identified as a recessive nuclear sterile plant based on infertility characteristics and cytological identification. The germplasm Stout is an early-maturing, high grain-yield, low groat protein-percentage, and short-strawed cultivar that was developed at Purdue University [48, 56]. The anther, microspore mother cells and microspores of CAMS line were imaged with the Olympus digital microscope. Both materials are highly adapted to the environment of northern China.

### RNA isolation

Total RNA was isolated using TRIzol reagent (Invitrogen) based on the manufacturer’s protocol, and mRNA was subsequently purified by oligotex (Qiagen). Total RNA samples were treated with DNase to degrade possible genomic DNA contamination. The ratios of A260/A280 and A260/A230 (1.8–2.0) were used to ascertain the RNA purity, which was assessed by 1.2% agarose gel electrophoresis using the RNA 6000 Pico LabChip Kit on the Agilent 2100 Bioanalyzer. Meanwhile, the ratio of 28S-to-18S rRNA (> 2) and the RNA integrity number (RIN) (RIN > 7) were calculated to evaluate RNA integrity using the Agilent 2100 Bioanalyzer.

### cDNA library preparation and transcriptome sequencing

The mRNA in RNA samples was enriched using magnetic beads conjugated with oligo (dT) and later degraded into short fragments at 94 °C in 5× fragmentation buffer (Illumina, USA). The synthesis of first-strand cDNA was performed by random hexamer primers, and second-strand cDNA was further synthesized in a reaction system with DNA ligase, DNA polymerase I, RNAse H, and dNTPs. Double-stranded cDNA was extracted using magnetic beads, and end repair and addition of nucleotide A (adenine) at the 3′ end were performed. Finally, the fragments were ligated with sequencing adapters and enriched by PCR amplification. The quality and quantification of libraries were evaluated using an Agilent 2100 Bioanalyzer and ABI StepOnePlus Real-Time PCR System, respectively. All libraries were sequenced on the BGISEQ-500 sequencing platform.

### Data processing of RNA-seq

To obtain clean reads, raw sequencing reads were filtered by removing low-quality reads with adapters, reads for which more than 20% of bases had qualities lower than 10, and reads with more than 5% unknown bases. Clean reads were assembled using Trinity (version 2.8.4) [23], and then RNA transcripts were clustered to generate unigenes by TIGR Gene Indices clustering tools (TGICL) [43]. To perform functional annotation for the unigenes, Blastn (version 2.2.23) and Blastx (version 2.2.23) [1] were used to align unigenes to Nucleotide (NT), nonredundant nucleotide (NR), EuKaryotic Orthologous Groups (KOG), Kyoto Encyclopedia of Genes and Genomes (KEGG) and SwissProt databases. Next, Blast2GO (version 2.5.0) [11] with NR annotation was used for Gene Ontology (GO) annotation, and InterProScan (version 5.11–51.0) [45] was employed for InterPro annotation. Transdecoder (version 3.0.1) (https://transdecoder.github.io) was used to identify the candidate coding regions of each unigene. The longest ORF (open reading frame) was extracted, and then the Pfam protein homologous sequences were searched by blast to SwissProt and Hmmscan to predict the coding regions. To identify transcription factors (TFs), ORFs of each unigene were predicted using getorf [47] and aligned to TF domains from PlntfDB [46] (http://plntfdb.bio.uni-potsdam.de) using hmmsearch [40].

Clean reads were mapped to unigenes using Bowtie2 (version 2.2.5) [31], and the fragments per kilobase of transcript per million base pairs sequenced (FPKM) value was subsequently calculated for each unigene using RSEM (version 1.2.12) [33]. Different expression analyses between the CMS line 722HA and maintainer line 722HB at different stages were performed by NOIseq [58]. Differentially expressed genes (DEGs) were defined by genes with probability ≥0.8 and | log2 fold change | ≥ 1 and were evaluated by hierarchical clustering using the pheatmap R package (version 1.0.12) [30]. Principal component analysis (PCA) was conducted using the PCAtools R package, and coexpression analysis was performed by MeV (version 4.9) (http://mev.tm4.org) with the k-means method. Hypergeometric tests of gene ontology and pathway functional enrichment for DEGs were performed using the Stats R package (version 3.7.0) [59]. GO functional enrichment analysis and KEGG pathway functional enrichment analysis were performed using phyper. The false discovery rate (FDR) of each *P* value was calculated, and the terms with FDR ≤ 0.01 were defined as significantly enriched. The target genes of transcription factors were confirmed by Minet, the Pearson R package, and the context likelihood of relatedness (CLR) method with a value ≥2.

### Quantitative real-time PCR for RNA-seq validation

Eleven sequences were selected randomly from the DEG list, and Primer Premier 5.0 software (PREMIER Biosoft International, CA, USA) was used to design the qRT-PCR primers (**Supplementary Table** **1**) with the following parameters: Tm 55 °C and primer length range of 16–22 nucleotides. The reaction system (in a total volume of 50 μL) of the Bio-Rad C1000 Thermal Cycler contained cDNA 4.0 μL, 20 mmol·L-1 PCR forward primer and reverse primer, SYBR® Premix Ex Taq II (Tli RNaseH Plus) fluorochrome 25.0 μL, and ddH_2_O 16.0 μL. PCR amplification was performed under the following conditions: 95 °C for 30 s followed by 40 cycles of 95 °C for 5 s, 55 °C for 30 s and 72 °C for 1:00, and fluorescence data were collected during the process. The housekeeping gene β-actin, which is usually used in gene expression experiments on plants, was selected as an internal control. PCR products were identified through 1.2% agarose gel electrophoresis.

## Results

### Morphological characteristics of the CAMS line

**Fig. 1:**
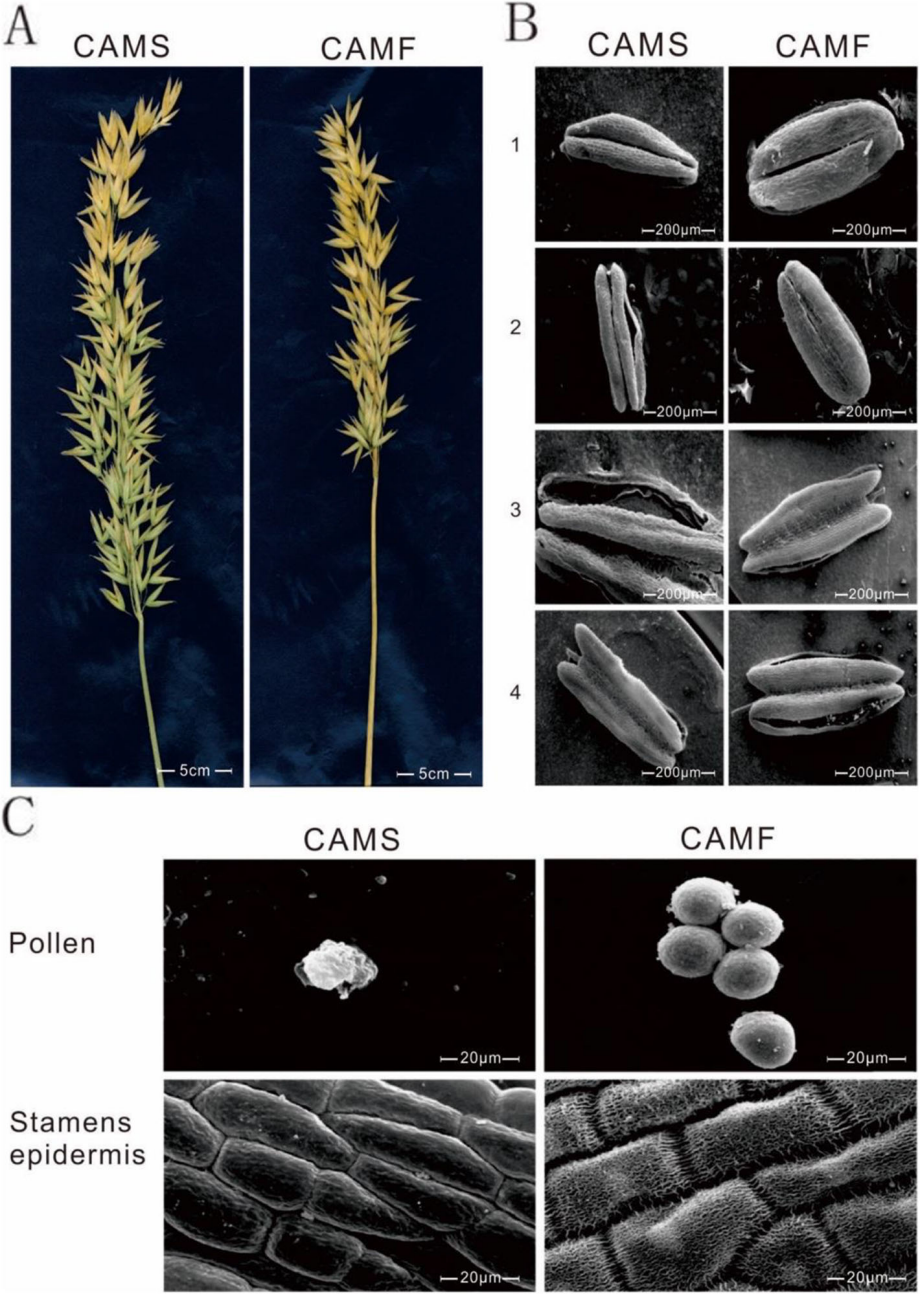
Plants, stamens, and pollen of the CAMS line and CAMF line. **a**. Plants of CAMS line and CAMF line. **b**. Electron micrograph of Stamens. CAMF, full of folds of the epidermis, with obvious pollen; CAMS, deflated, and no corrugation on the surface. **c**. Pollen: CAMF, oval, good activity; CAMS, deformity, and inactive

**Fig. 2:**
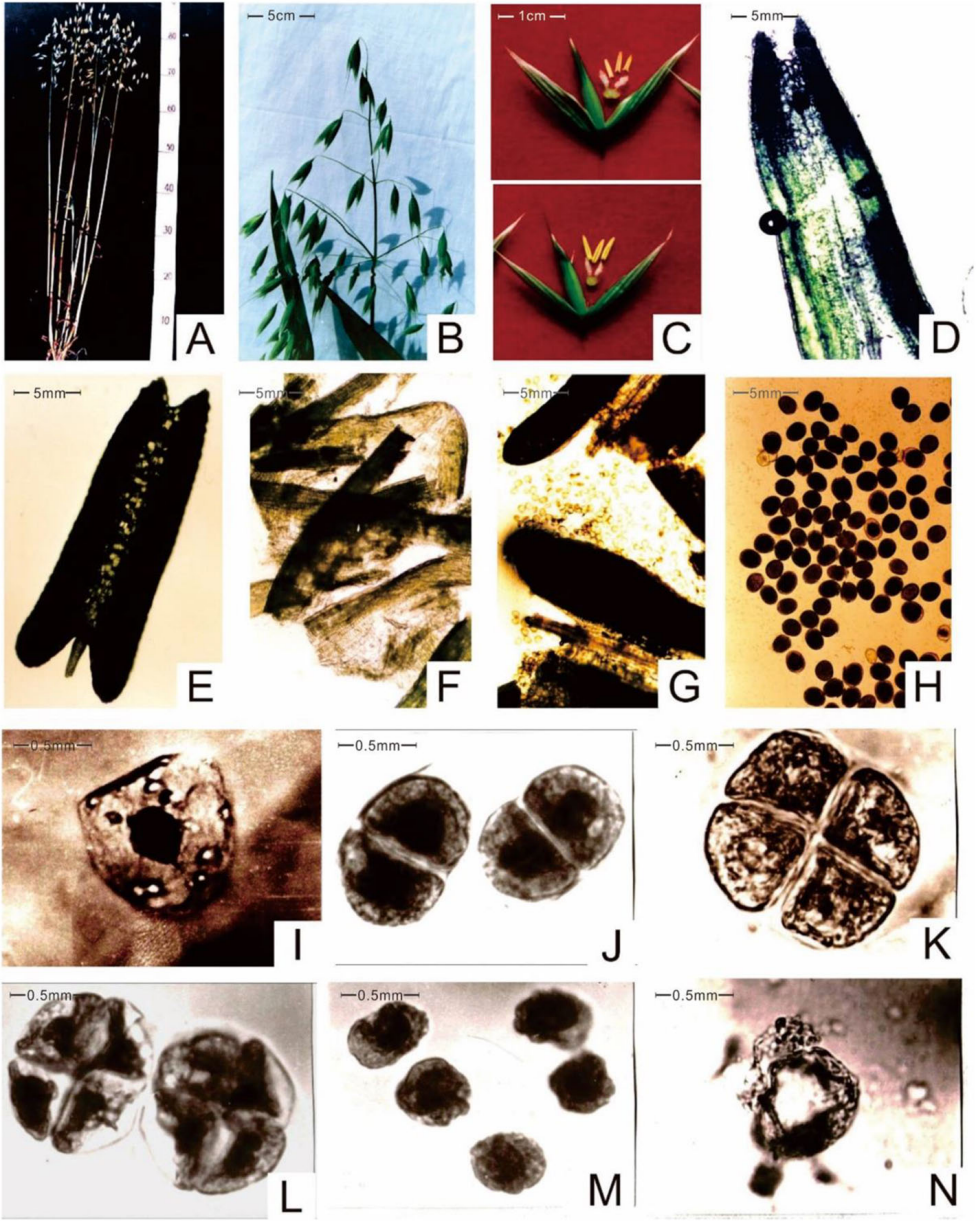
Phenotypic characterization of CAMS line. **a**, Phenotype of oat. **b**, Regulate panicle of oat. **c**, Stamen of CAMS line (left) and CAMF (right). **d-h**, Anther of CAMS line. I-K, Microspore mother cells of CAMS line. **l**, Degenerated cytoplasm of microspore mother cells for the CAMS line. **m-n**, Microspores shape of CAMS line

The CAMS material used in this study was first reported by Cui Lin in the oat testing field of the Alpine Crops Research Institute in 1994, and the fertile material utilized in the same field was named CAMF [12] (Fig. 1). The oat takes root and grows straight upward to 80–90 cm in height over approximately 85 days (Fig. 2**a**), exhibiting dark green leaves and compact main stems, and it produces a number of tillers at the bases of stems (Fig. 2**b**). The number of tillers/plants is usually approximately three to five, and the short stem terminates in a regulated panicle that is arranged in a spikelet at approximately 18 cm. Within the panicle, the average number of spikelets per spike was 28, and each spikelet had three flowers (Fig. 2**c**). There is a specific short awn approximately 2 cm located on the outside of the second flower, and the glume turns yellow after ripening. All these traits of the CAMS line are the same as those of the CAMF line in the same population.

In contrast to the CAMF line, the glume of the CAMS line opens at the first 3–5 days of flowering, and the light green anthers of the CAMS line are smaller than those of the CAMF line. The filaments of CAMS stamens elongate during the 7–10 days of flowering, the anthers do not crack and shrink into an arrow shape with gray color (Fig. 2**c**), and the pistils are normal and in keeping with the CAMF line. Compared with the opaque anthers of sterile plants filled with normal pollen grains, the transparent anthers of CAMS were not filled with pollen grains when observed under the microscope, indicating that the CAMS line belongs to the nonpollen type (Fig. 1**b-c,** Fig. 2**d-h**).

### No pollen detected in anthers of the CAMS line

To understand the differences between CAMS and CAMF lines regarding the meiosis process of microspore mother cells, the pollen grains of these two lines were collected from stamens at between 7 and 8 a.m. The smearing showed that the CAMS and CAMF lines could form normal microspore mother cells by mitosis and tetrads by meiosis. Compared with the CAMF line, the CAMS line was normal in the early stage of tetraspores (Fig. 2**i-k**), while the significantly degenerated cytoplasm of microspore mother cells for the CAMS line was observed in the late stage (Fig. 2**l**). The microspores released by the tetraspore of the CAMS line did not continue to develop on their outer walls, which were thicker than normal and mostly deformed with a few rounds (Fig. 2**m-n**). The cell wall of the CAMS line decomposed at the later stage of pollen development and exhibited empty and nonpollen characteristics.

### Male sterility inherited by recessive nuclear genes

To investigate genotypes of the CAMS line, crossing between CAMS and CAMF lines was performed, showing that all F1 plants were fertile (Table 1). Some sterile plants appeared in the F2 population in the greenhouse and field, indicating that environmental factors, such as temperature and light, do not affect fertility. However, no fertility separation was found in the CAMF line of the original population used for pollination, indicating that the CAMF line used for pollination was homozygous. The results indicate that the infertility traits of CAMS are stably inherited and do not belong to thermosensitive or light-sensitive infertility.

**Table 1:** Expression of male sterility in different places

Combination		Place	Number of plants	Number of fertile plants	Number of sterile plants
Sterile × Fertile	F1	Green house	54	54	0
	F1	Field	39	39	0
	F2	Field	312	233	79
	F2	Green house	249	185	64
	F2	Field	180	136	44
CAMS		Field	289	289	0

Furthermore, the CAMS line hybridized with eight varieties of *A. chinensis* and *A. sativa*. Among the F1 populations, the fertility of six combinations was fully restored with normal seed setting rates (Table 2), and two combinations showed approximately 20% complete sterile lines. Next, the rate of sterile BC1 and BC2 plants increased when the two hybrid combinations were selected to continue to backcross with their parents (Table 3). However, the sterile plant rate of BC1 and BC2, with *A. chinensis* 8006 serving as the parent, was higher than that of *A. sativa* 9203. When the CAMS line hybridized with other varieties, the reproductive ability was restored, and sterile plants of the F1 generation appeared, indicating that the inheritance of CA sterility traits in oats is not only related to nuclear sterility genes but is also affected by nuclear restorer genes. All F1 plants from the combinations of fertility restoration were selected for selfing to generate F2 plants. F2 progeny showed a 3:1 segregation ratio of CAMF and CAMS plants (Table 4), suggesting that male sterility is controlled by a pair of recessive nuclear genes. The test cross was performed using the F1 plant and CAMS line to generate the BC1 population. BC1 generation displayed a 1:1 segregation ratio, which is consistent with the principles of traits controlled by nuclear genes (Table 5).

**Table 2:** Fertility of F1 progeny from the cross of the CAMS line and other varieties

Combination	No. of plants	No. of fertile plants	No. of sterile plants	% of sterile plants
CAMS× 8024 (*A. sativa*)	68	68	0	0
CAMS× 8028 (*A. sativa*)	123	123	0	0
CAMS× 8869 (*A. sativa*)	89	89	0	0
CAMS×9203 (*A. sativa*)	116	92	24	20.7
CAMS× 7818 (*A. chinensis*)	61	61	0	0
CAMS× 6618 (*A. chinensis*))	97	97	0	0
CAMS× 6405 (*A. chinensis*))	74	74	0	0
CAMS×8006 (*A. chinensis*))	101	78	23	22.8

**Table 3:** Backcross results of the CAAMS line in two combinations

Combination		Number of plants	Number of fertile plants	Number of sterile plants	% of sterile plants
CAMS× 6902(*A. sativa*)	BC1	60	44	16	26.7
CAMS×6902(*A. sativa*)	BC2	69	46	23	33.3
CAMS× 6800(*A. chinensis)*	BC1	42	26	16	38.1
CAMS×6800(*A. chinensis*)	BC2	58	22	36	62.1

**Table. 4:** Fertility of F2 progeny from the cross of the CAMS line and other varieties

Combination	No. of plants	No. of fertile plants	No. of sterile plants
CAMS×8024(*A. sativa*)	358	269	89
CAMS×8028(*A. sativa*)	356	268	88
CAMS×8869(*A. sativa*)	49	348	111
CAMS×7818(*A. chinensis*)	311	236	75
CAMS×6618(*A. chinensis*)	428	327	101
CAMS×6405(*A. chinensis*)	487	368	119

**Table 5:** Fertility segregation of F1 progenies by crossing the CAMS line and other varieties

Combination	No. of plants	No. of fertile plants	No. of sterile plants
CAMS×(CAMS×CAMF)F1 CAMS×Heterozygous	141	69	72
CAMS×(CAMS×8024)F1 CAMS Heterozygous	234	115	119
CAMS×(CAMS×8028)F1 CAMS×Heterozygous	237	121	116
CAMS×(CAMS×8869)F1 CAMS×Heterozygous	130	62	68
CAMS×(CAMS×7818)F1 CAMS×Heterozygous	94	46	48
CAMS×(CAMS×6618)F1 CAMS×Heterozygous	135	64	71
CAMS×(CAMS×6405)F1 CAMS×Heterozygous	99	51	48

### Cultivation of the near-isogenic line

**Fig. 3:**
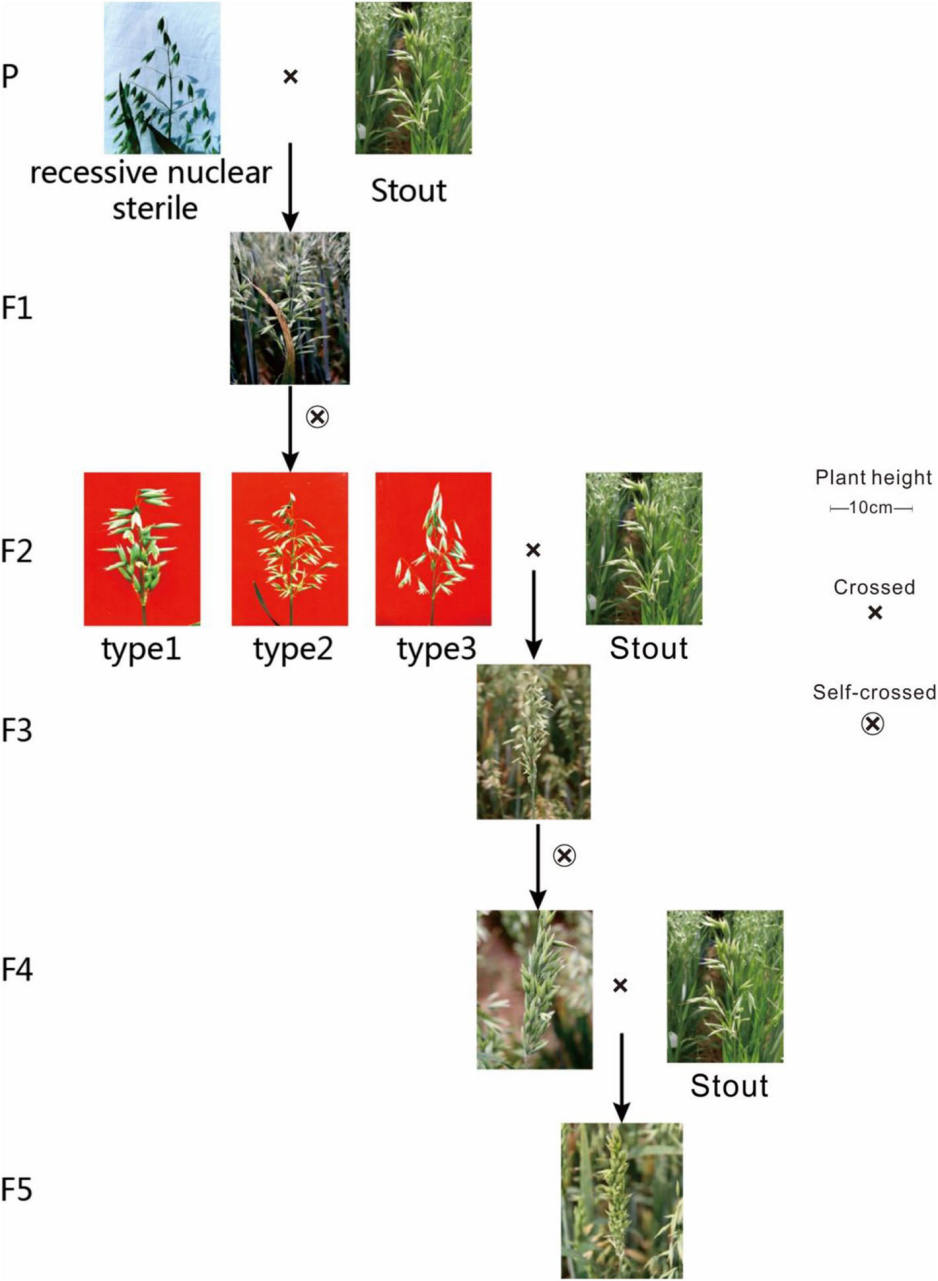
Breeding process of oat BC5F8

To transform the original spikelets of sterile plants downward, a near-isogenic oat line was generated by iterating recombinant inbred material (*A. sativa* L., 6n = 42, ACD). The sterile oat plant was crossed with oat germplasm Stout to generate F1 and F2 (Fig. 3). After that step, the sterile F2 material was backcrossed with Stout to generate fertile BC1. Next, BC1s were self-crossed to generate BC1F2. After that cross, the sterile BC1F2 plant was backcrossed with Stout to obtain BC2F1. Using a protocol similar to that described above, we obtained BC2F1, BC3F1, BC4F1 and BC5F1. Finally, we harvested BC5F8 cells using continuous self-crossing methods.

We carried out potted planting for BC5F8 at 20 °C, 65 ± 20% relative humidity, 12 h illumination per day and 4000 lx light intensity. We selected five spikes when the length of the spikelet was 0–5 cm. We sampled anthers of different developmental stages in accordance with their size and color. The tissue samples were immediately frozen in liquid nitrogen and stored at − 80 °C.

### Anther samples collection and sequencing

To identify the genes that play critical roles in CAMS pollen abortion, we performed time-course RNA sequencing on anthers of the CAMS line and CAMF line. Samples were collected based on the timing of morphological, anatomical, physiological and cytological characteristics that occur during the genesis of pollen in the stamen of oat (Table 6). The anthers of CAMF and CAMS were selected when the length of the spikelet was 0–5 cm. The stamen developmental stages were named F1, F2, F3, and F4 and S1, S2, S3, and S4 for CAMF and CAMS, respectively. The sampling time of spikelets was 9 a.m. There were four stages used in our experiments, specifically white stamen (stage 1, CAMS and CAMF), green stamen (stage 2, CAMS and CAMF), green turning to yellow stamen (stage 3, CAMS and CAMF), light yellow stamen (stage 4, only for CAMS line), and dark yellow stamen (stage 4, only for CAMF) (Table 6). Samples were immediately frozen in liquid nitrogen and stored at − 80 °C for 30 min. The total RNA of CAMS and CAMF was extracted, and two paired-end cDNA libraries were constructed for RNA sequencing (RNA-seq). Meanwhile, the total RNA of each sample (stages 1 to 4 for CAMS and CAMF) was extracted to generate a cDNA library for signal-end RNA sequencing. In total, 24 cDNA libraries were separately constructed. Sativa anther samples of the CAMS and CAMF lines were sequenced on the BGISEQ-500 platform.

**Table 6:** Comparison of staminal morphology between CAMS and CAMF liens

Stages	Fertile		Sterile	
	Sample	Phenotype	Sample	Phenotype
Stage 1	F1	White	S1	White
Stage 2	F2	green	S2	green
Stage 3	F3	Green turns to yellow	S3	Green turns to yellow
Stage 4	F4	Dark yellow	S4	Light yellow

### Transcriptome assembly and annotation

A total of 943.06 M and 932.54 M raw sequencing reads were obtained for CAMF and CAMS samples, respectively. After filtering, 846.05 M high-quality reads with 96.85% Q20 percentage and 839.35 high-quality reads with 97.16% Q20 percentage were obtained for CAMF and CAMS samples, respectively. Clean reads were assembled de novo into 295,462 unigenes with an average length of 1511.8 bp, of which 230,590 unigenes (78.04%) were longer than 500 bp, and 173,160 unigenes (58.61%) were longer than 1000 bp.

All unigenes were aligned against the nonredundant (NR), nucleotide collection (NT), Gene Ontology (GO), Swiss-Prot, Kyoto Encyclopedia of Genes and Genomes (KEGG), and Clusters of Orthologous Groups of proteins (COG) databases, and there were 191,958 (64.97%), 162,676 (55.06%), 139,482 (47.21%), 134,608 (45.56%), 146,769 (49.67%), and 137,657 (46.59%) unigenes annotated in these databases, respectively. A total of 210,003 (71.08%) unigenes were annotated in one or more of these six databases. Transcription factors and TF families were further analyzed, which showed that MYB (612 genes; 12.13%) was predicted as the largest TF family followed by bHLH (354 genes; 7.02%), AP2-EREBP (328 genes, 6.5%), and NAC (319 genes; 6.32%) (**Supplementary Table** **2**).

To identify candidate male sterility genes that might lead to anther male sterility, we also evaluated transcripts with the Plant MicroRNA Database (PMRD) (Cui, 2012). Four transcripts were identified by VLAST and were highly similar to five genes (six transcripts) of *Arabidopsis thaliana*. Meanwhile, 82 transcripts were identified as homologous sequences with 77 transcripts of *Oryza sativa*.

### FKPM calculation and DGEs detection

**Fig. 4:**
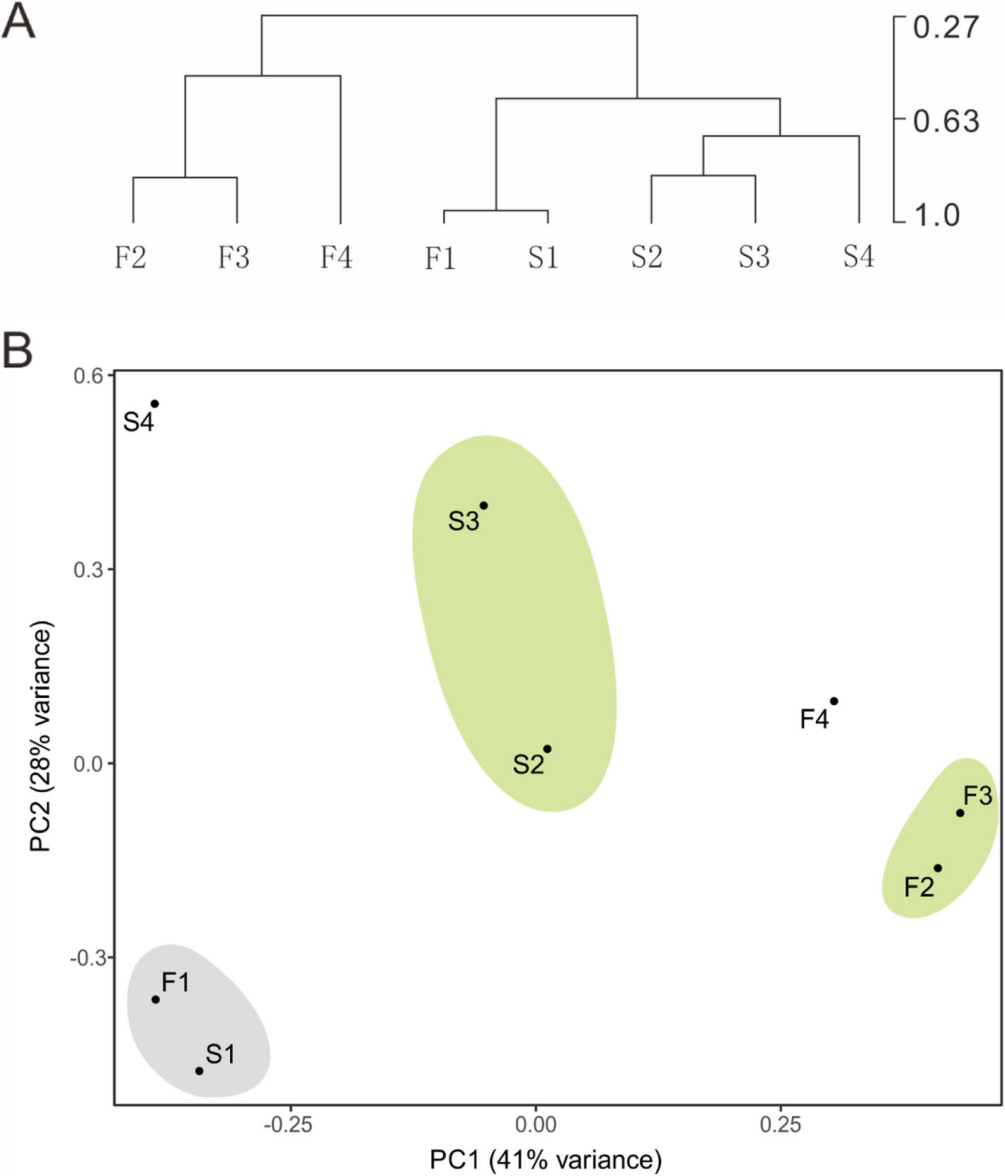
HCL **a** and PCA **b** analysis of all developmental stages

FPKM values of all samples were calculated based on the assembled transcriptomes, and a total of 281,944 genes with FPKM values larger than zero in at least one sample were contained. The correlation coefficients of different samples at the same stage were all ≥0.9; therefore, the average expression value of the same stages was used as the representative expression value for that stage, and 26,866 genes had an expression level ≥ 10 in at least one sample. According to the time course of development, DEGs were identified between every two adjacent stages for the same samples and between every two samples for the same stages, and 217,477 DEGs were identified with fold change ≥2.00 and probability ≥0.8 (**Table** **7****and** Fig. 4). Among these DEGs, 48,213 and 26,866 genes had an expression level larger than 5 and 10 in at least one sample, and the number of DEGs with expression ≥10 in at least one stage accounted for more than 90% of the total. To reduce the impact of low-expression genes, the DEGs with expression ≥10 in at least one group were chosen for further analysis.

**Table 7:** Statistical summary DEGs between varieties and throughout the developmental stages

Categories	Up-regulated DEGs	Down-regulated DEGs	Total DEGs
F1-vs-F2	34894	47827	82721
F1-vs-F3	38372	54627	92999
F1-vs-F4	37367	92639	130006
F1-vs-S1	9504	17700	27204
F2-vs-F3	20158	19537	39695
F2-vs-F4	30437	67650	98087
F2-vs-S2	36088	16494	52582
F3-vs-F4	19840	51936	71776
F3-vs-S3	57764	31323	89087
F4-vs-S4	91154	39681	130835
S1-vs-S2	45550	26932	74282
S1-vs-S3	50859	34285	85144
S1-vs-S4	54393	48597	102990
S2-vs-S3	24688	28676	53364
S2-vs-S4	42191	52768	94959
S3-vs-S4	22121	34290	56411

### Sterility of the CAMS line occurs in the early stage of stamen development

Smearing showed that the tetrad of the CAMS line was normal and in keeping with the CAMF line in the early stage of stamen development, while significant cytoplasmic degradation of microspore mother cells for the CAMS line was observed in the late stage. For further detection, we performed hierarchical clustering and principal component analysis (PCA) analysis on 28,942 genes, showing that the expression patterns of CAMS and CAMF in the first stage were very similar, and there were clear differences between them at the beginning of the second stage. CAMS and CAMF showed completely different development patterns in the second and third stages separately and exhibited clear differences in the fourth stage (Fig. 4), confirming that the sterility of the CAMS line occurred in the early stage of stamen development.

### Sterility of the CAMS line related to meiosis and superoxide

**Fig. 5:**
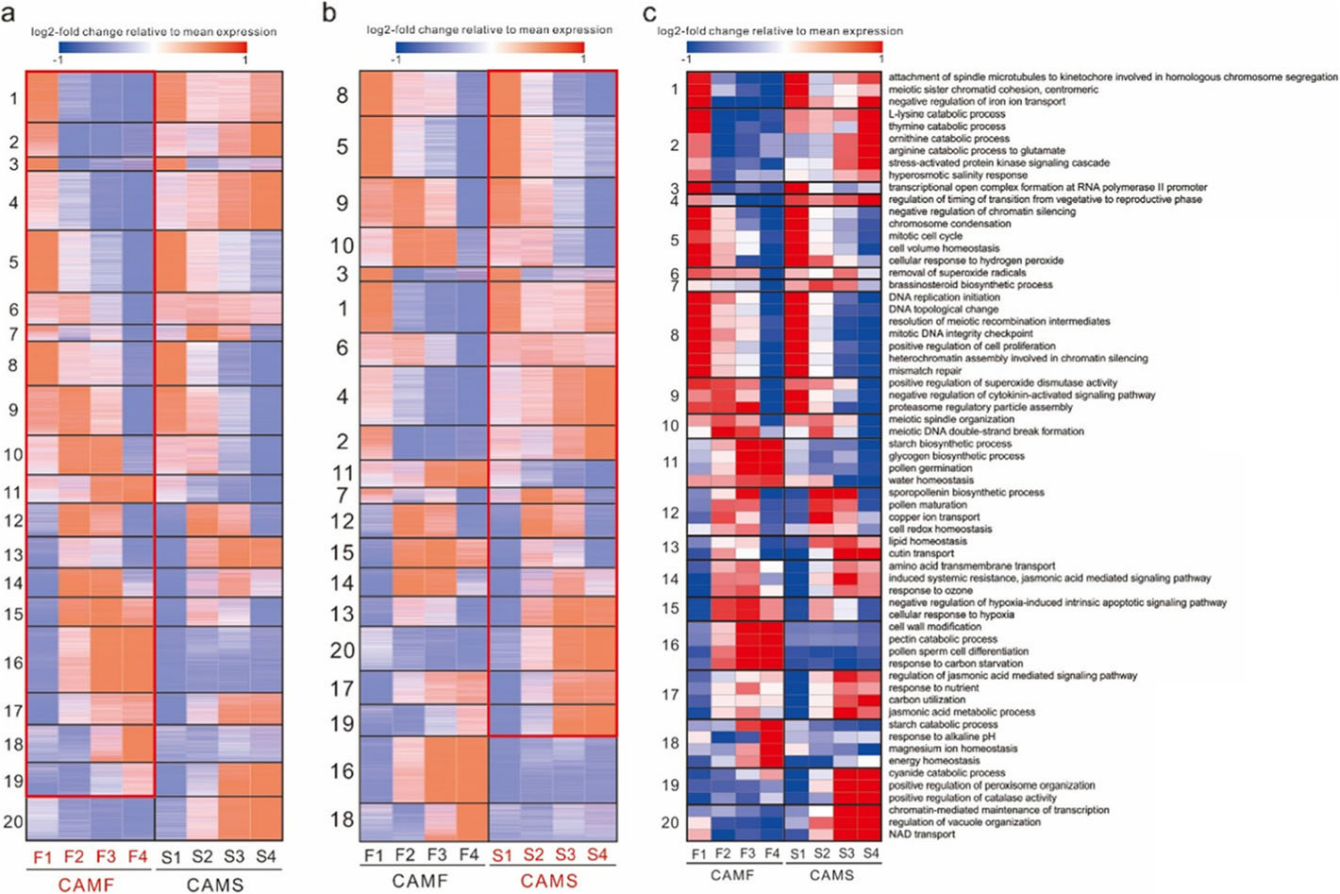
K-means heatmap and functional enrichment of the corresponding gene set

To further determine the factors responsible for the sterility of the CAMS line, the expression of 28,942 genes was transformed with log2 (FPKM+ 0.01) and standardized by z-score. K-means clustering was performed on these genes, and they were clustered into 20 coexpression modules, of which the gene expression was determined by the average z-score value of all contained genes, and GO enrichment was performed for each module after clustering. The enriched GOs were quantified by GO annotation results and z-score normalized FPKM of 28,942 genes, and the GO expression was calculated by the average z-score of the containing genes. Finally, correlation analysis of the weighted gene coexpression network analysis (WGCNA) was performed for each module and each GO, and 354 GO BP terms were maintained after filtering with 0.9 of the correlation coefficient (Fig. 5**and Supplementary Table** **3**).

**Fig. 6:**
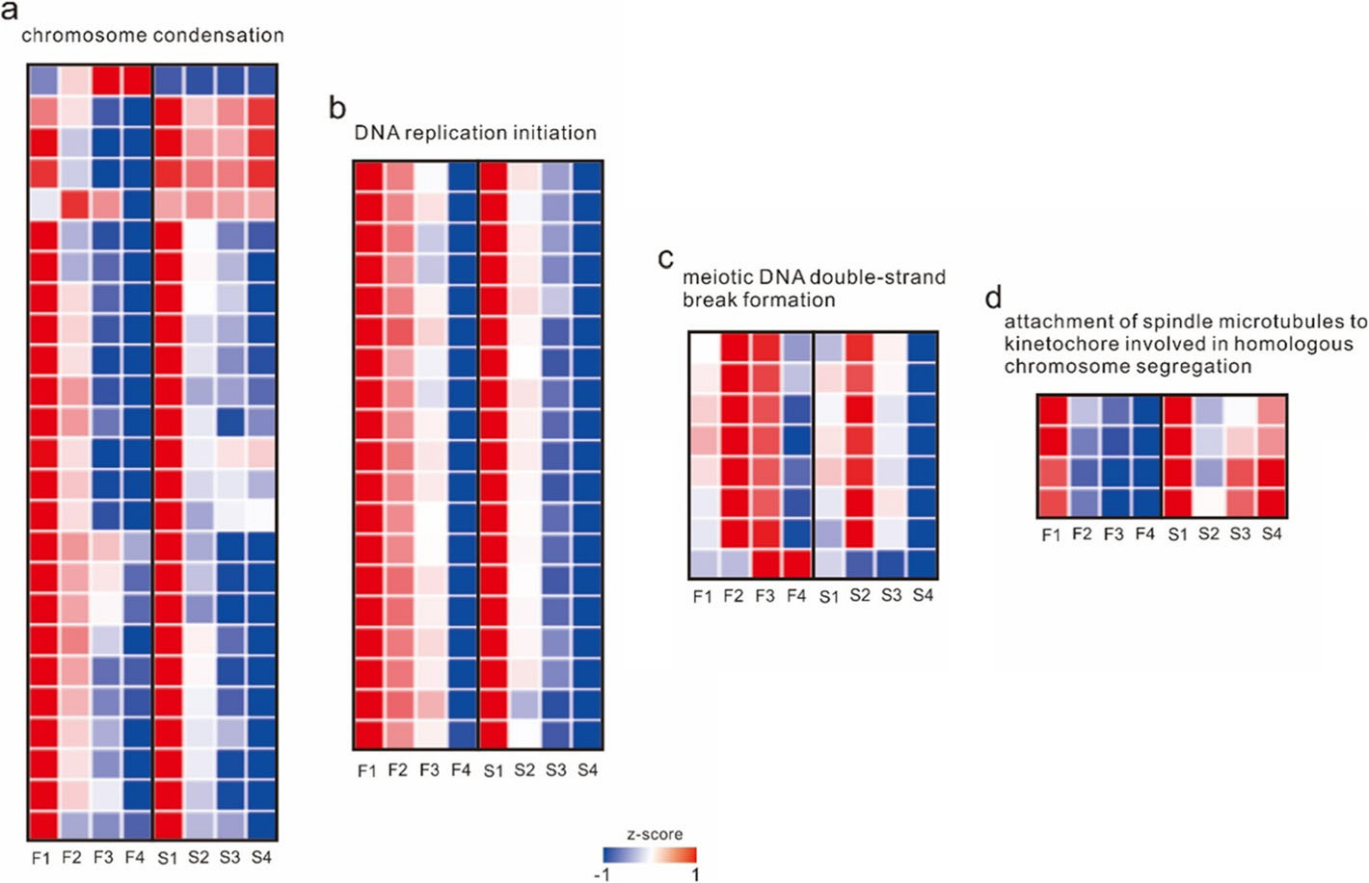
Heatmap of expression levels of meiosis-related genes

Meanwhile, the possible target genes of transcription factors were estimated by context likelihood of relatedness (CLR) algorithms. Eleven modules were determined to be highly expressed in the early stage, of which four modules were related to deceleration and splitting, including modules 1, 5, 8 and 10. Module 1 was significantly enriched in the attachment of spindle microtubules to kinetochores involved in homologous chromosome segregation and meiotic sister chromatid cohesion and centromerics. Module 5 was significantly enriched for chromosome condensation, and module 8 was enriched for DNA replication initiation and resolution of meiotic recombination intermediates. Module 10 was significantly enriched in meiotic spindle organization and meiotic DNA double-strand break formation (Fig. 5**and Supplementary Table** **3**). Combining the modules’ functions and the genes’ FPKM, the FPKM of CAMS was similar to that of CAMF in the first developmental stage, while they were significantly different in the second stage. After accumulating the FPKM of the four stages, the whole FPKM of genes related to chromosome condensation and the attachment of spindle microtubules to kinetochores involved in homologous chromosome segregation of the CAMS line was higher than that of the CAMF line, while the expression levels of genes related to DNA replication and meiotic DNA double-strand break formation in the CAMS line were lower. In addition, genes related to chromosome condensation and attachment of spindle microtubules to kinetochores involved in homologous chromosome segregation were highly expressed in all developmental stages in the CAMS line, while they were only highly expressed in the first and second stages in the CAMF line. The expression of genes related to DNA replication and meiotic DNA double-strand break formation in the CAMS line was significantly lower than that in the CAMF line in the second and third stages (Fig. 6**and Supplementary** Table 4), and the most related transcription factors to these genes were MADS and Alfin-like (**Supplementary Table** **5**). According to these modules, the direct reason for the sterility of CAMS was abnormal meiosis after the second stage, and module 5 also included genes related to the cell’s response to hydrogen peroxide, which were expressed at higher levels in the CAMS line, indicating that the cells of the CAMS line may contain more hydrogen peroxide.

There was no significant difference in the expression of genes related to sporopollenin biosynthesis (module 12) between the CAMS and CAMF lines, but the genes related to starch synthesis and glycogen synthesis (module 11 and module 16) maintained high expression in all stages of the CAMF line, which supplied energy for meiosis and pollen development, and these genes were only highly expressed in the early stages of the CAMS line. In the middle and late developmental stages, the expression of genes related to resistance and stress response in the CAMF line (module 14) was higher than that in the CAMS line. Resistance mainly induced systemic resistance, and the stress response was primarily utilized in the signaling pathway mediated by jasmonic acid. In addition, the CAMS line was more sensitive to hypoxia than the CAMF line. Genes related to negative regulation of hypoxia-induced intrinsic apoptosis signaling pathways and cell response to hypoxia (module 15) had high expression in the middle and late stages of the CAMF line, while in the CAMS line, there was only a detectable level of expression in the middle stage. The meiosis of the CAMS line was abnormal in the second stage, inducing the CAMS line to progress in the middle and later stages, such as the regulation of the transition time from the vegetative stage to the reproductive stage and genes related to brassinosteroid synthesis with higher expression in the middle and late stages (Fig. 5**and Supplementary Table** **3**).

**Fig. 7:**
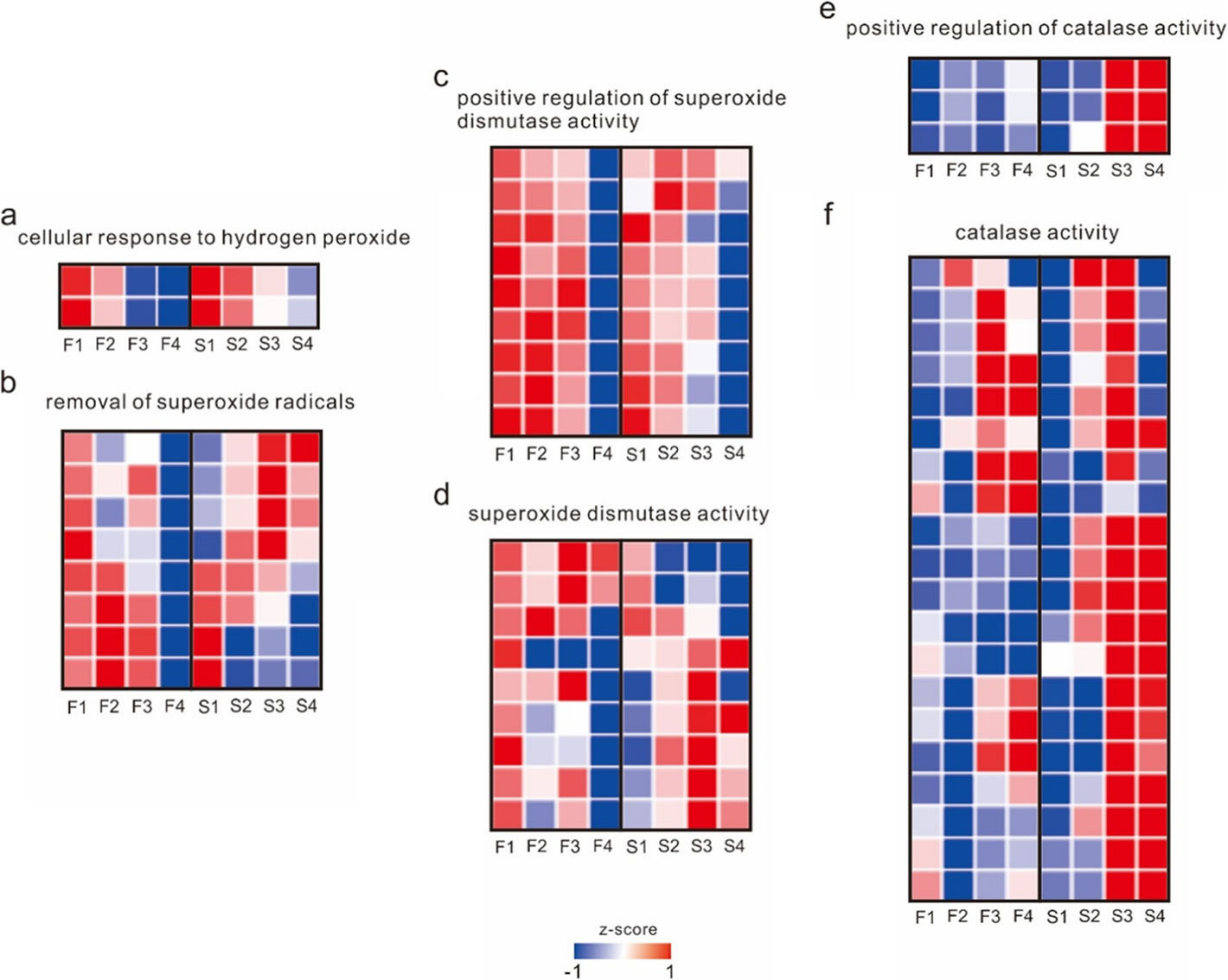
Heatmap of the expression levels of superoxide-related genes

There were five modules related to superoxide, including module 5, significantly enriched in cellular response to hydrogen peroxide; module 9, enriched in positive regulation of superoxide dismutase activity; module 6, enriched in removal of superoxide radicals; module 15, enriched to negative regulation of hypoxia-induced intrinsic apoptotic signaling pathway; and module 19, significantly enriched to positive regulation of catalase activity (Fig. 5**and Supplementary Table** **3**). Combining the expression of all genes corresponding to these functions, the expression patterns of these genes were different from those of meiosis-related genes, and the gene expression was most different in the first stage between the CAMS line and CAMF line. The expression of genes related to the positive regulation of superoxide dismutase activity, superoxide dismutase activity, catalase activity, and removal of superoxide radicals in the CAMS line was higher than that in the CAMF line in the first stage, and the expression of genes related to superoxide dismutase activity and removal of superoxide radicals in the CAMS line was higher in the first three stages. In general, the gene expression of the CAMS line in the last two stages was higher than that in the first two stages. Genes related to the cellular response to hydrogen peroxide were highly expressed in the first two stages in the CAMS line, while they were highly expressed in all stages in the CAMF line. Genes related to the positive regulation of catalase activity in the CAMS line were highly expressed and significantly higher than those in the CAMF line in the last two stages (Fig. 7**and Supplementary** Table 4). The most relevant transcription factors for these transcripts were Tify and MADS (**Supplementary Table** **5**). In summary, the superoxide scavenging ability of the CAMS line was lower than that of the CAMF line in the early stage, while it was different in the late stage. The lack of oxide scavenging ability caused the accumulation of superoxide in the cell and affected meiosis, while the CAMS line accelerated the scavenging ability of superoxide, which meant that stamens could continue to develop in the late stage.

### qPCR confirmations

**Fig. 8:**
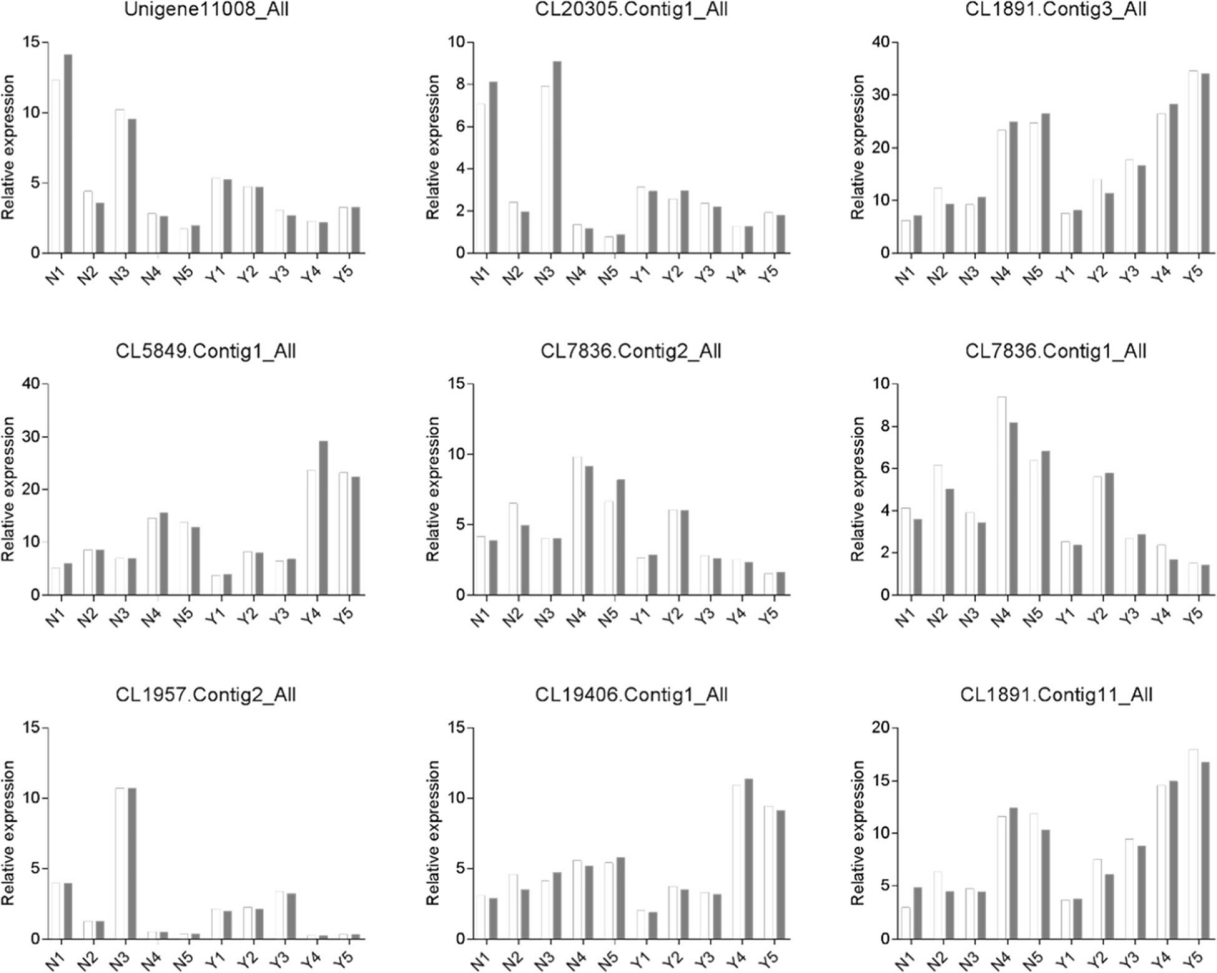
Quantitative real-time PCR (qRT-PCR) validation of nine DEGs. White and gray bars represent the expression results of RNA-Seq analysis and qRT-PCR, respectively

To confirm the transcriptome sequencing analysis results, qRT-PCR confirmation was employed in this study. For accurate and reliable results, nine genes were selected randomly (Fig. 8), and the expression patterns of nine candidate genes were validated using the qRT-PCR normalization method. The expression patterns of most candidate genes in the qRT-PCR results were consistent with those in the RNA sequencing analysis results, proving the reliability of our sequencing analysis.

## Discussion

A complex mechanism governs male fertility from the initial morphogenesis to the release of flower pollen by anther dehiscence. Pollen development is a complex process involved in the development, differentiation, meiosis, cell cycle and morphogenesis of anthers and involves several tissues and cell types. Meiosis is the first phase for spore formation and pollen development [21]. The dysfunction of gene products associated with meiosis induced male sterility [3, 34, 42].

The plant pollen wall plays crucial roles in the development of pollen, and mature pollen is released from anther dehiscence. Dysfunction in the pollen cell wall could induce male sterility [50, 53]. The endothecial cells of stamens deposit lignin using the phenylpropanoid biosynthesis pathway [14, 22], which contributes to wall thickening and anther dehiscence [51]. Sporopollenin, cutin, suberin, and lignin are the primary structural components of the pollen wall [51], and the secretory tapetum deposits carotenins, flavonols, and lipids onto the exine surface of the pollen wall [49].

After meiosis, microspores are enveloped on a callose wall with β-1,3-glucan and a pollen mother cell (PMC) wall with cellulose, hemicelluloses and esterified pectin [50]. The pollen wall consists of several layers of chemically different materials [37] and is important in angiosperm reproductive development [24], and most sterile mutants are caused by pollen wall defects [53]. Therefore, the formation and degradation of these cell walls are important for pollen development [50]. In our study, many DEGs were enriched in GO terms related to the cell wall and extracellular region.

Previous studies have shown that pectinesterase could convert the de-esterification of protopectin to soluble pectin and methanol [36], and this enzyme is also essential for the modification of the cell wall and the growth of pollen tubes [5, 6, 18, 26]. In this study, the DEGs were also enriched in pectinesterase activity-associated GO terms. Callose, a cell wall polysaccharide composed of 1,3 β-glucans, plays an important role in the formation of the cell wall [16, 44, 60], and the abnormal metabolism of callose during microsporogenesis induces degeneration of microspores and male sterility [17, 32]. In our study, DEGs were enriched in the GO terms polysaccharide catabolic process and glucan catabolic process, indicating that the dysfunction of the staminal cell wall might be an important reason for male sterility in oats.

Myosin and actin promote the growth of pollen tubes, which are closely associated with the plasma membrane and cell projections [8, 19, 61, 62]. Meanwhile, many DEGs were determined to be enriched in membrane-like structure and myosin; therefore, the genes related to membrane and myosin were defective in the development of anthers of the CAMS line.

Carbohydrates play important roles in the development of anthers and pollen [20], and alterations in carbohydrate metabolism or assimilate supply are involved in pollen abortion [15]. Compared with the CAMF line, the sugar levels in the anthers of male sterile Indian mustard lines were significantly lower [4]. As a characteristic of male sterile lines, changes in carbohydrate levels and enzyme activities could decrease sugar and starch accumulation in the anthers [4, 52]. Therefore, the categories of transport and carbohydrate metabolism were overrepresented among DEGs in CAMS, indicating that cellular material transport and carbohydrate metabolism might be inhibited in the development of CAMS anthers.

The development of anther and male gametophytes is regulated by complex gene expression patterns [38, 51]. As the inner layer of the anther wall, the tapetum shows high metabolic activity, which could satisfy necessary nutrients for pollen development [51, 57], and it is also related to male sterility [67]. Genes involved in protein, starch, and sucrose metabolism, osmoregulation, sugar transport, lipid transfer, flavonoid synthesis, and cytoskeleton structure were identified in this research [51].

In addition, we also explored candidate genes for male sterility by evaluating transcripts with the Plant MicroRNA Database (PMRD) database [13]. Four transcripts were identified by VLAST and were highly similar to five genes (six transcripts) of *Arabidopsis thaliana*. Among these genes, CDC48, cell division cycle 48, encodes a cell division control (CDC) protein and is reported to have functions in pollen germination and pollen tube growth [39]. Meanwhile, 82 transcripts were determined to be homologous with 77 transcripts of *Oryza sativa*, and among these transcripts, PTC1, OsGSL5, and Ugp2 were related to male sterility in plants. PTC1, persistent tapetal cell1, is required for tapetal cell death and pollen development in rice [1]. OsGSL5, encoding the callose synthase 1 catalytic subunit, might play an essential role in callose synthesis during microsporogenesis [66]. UDP-glucose pyrophosphorylase 2 (OsUgp2), a pollen-preferential gene in rice, plays a critical role in starch accumulation during pollen maturation [41].

## Conclusion

The cross-backcross test proved that the traits of the CAMS line were controlled by recessive nuclear genes. The results of PCA and HCL analysis showed that the expression patterns of the CAMS line and CAMF line were very similar in the early stage of stamen development and differed at the beginning of the second stage, indicating that the sterility of the CAMS line occurred in the early stage of stamen development, which was also in keeping with the smearing results. The functions related to meiosis and superoxide were significantly enriched by multiple modules in the K-means clustering, and the expression level of meiosis-related genes was not significantly different in the first stage, while it was significantly different after the second stage. Meanwhile, the expression patterns of genes related to the hydrogen oxide reaction were the same as those of meiosis-related genes, indicating that the sterility of the CAMS line was primarily caused by abnormal meiosis and related to superoxide. We further found that the expression of genes related to the superoxide scavenging ability of the CAMS line was lower than that of the CAMF line in the early stage, while it was higher in the CAMS line in the late stage, showing that the superoxide scavenging ability of the CAMF line was higher than that of the CAMS line in the early stage and that the CAMS line was higher in the late stage. Due to the insufficient superoxide scavenging ability of the CAMS line, superoxide accumulated in cells and affected cell meiosis. However, in the late stage, the scavenging ability of superoxide in the CAMS line was accelerated such that stamens could develop.

## Supplementary Information

**Additional file 1: Supplementary Table 1.** Primer sequences and their product size in qRT-PCR.

**Additional file 2: Supplementary Table 2.** Annotation of transcription factors for unigenes.

**Additional file 3: Supplementary Table 3.** GO BP enrichment of different clusters by K-means clustering.

**Additional file 4: Supplementary Table 4.** Expression levels of meiosis-related genes and superoxide-related genes.

**Additional file 5 Supplementary Table 5.** Target genes in transcription factors based on the CLR method.

## Data Availability

All data and materials mentioned in this article are available. Sequencing reads have been submitted to the NCBI Sequence Read Archive (SRA) under accessions SRR11431571-SRR11431594.

## Abbreviations

MS: Male sterility
CMS: Cytoplasmic male sterility
GMS: Genic male sterility
GCMS: Genic-cytoplasmic male sterility
CAMS: CA male sterile
CAMF: CA male fertile

## References

[1] Altschul SF, Gish W, Miller W, Myers EW, Lipman DJ (1990). Basic local alignment search tool. J Mol Biol.

[2] Anderson JW, Ward K (1979). High-carbohydrate, high-fiber diets for insulin-treated men with diabetes mellitus. Am J Clin Nutr.

[3] Azumi Y, Liu D, Zhao D, Li W, Wang G, Hu Y, Ma H (2002). Homolog interaction during meiotic prophase I in Arabidopsis requires the SOLO DANCERS gene encoding a novel cyclin-like protein. EMBO J.

[4] Banga S, Labana K, Banga SK (1984). Male sterility in Indian mustard (Brassica juncea (L.) Coss.)—a biochemical characterization. Theor Appl Genet.

[5] Bosch M, Cheung AY, Hepler PK (2005). Pectin methylesterase, a regulator of pollen tube growth. Plant Physiol.

[6] Bosch M, Hepler PK (2005). Pectin methylesterases and pectin dynamics in pollen tubes. Plant Cell.

[7] Braaten J, Wood P, Scott F, Wolynetz M, Lowe M, Bradley-White P, Collins M (1994). Oat beta-glucan reduces blood cholesterol concentration in hypercholesterolemic subjects. Eur J Clin Nutr.

[8] Cárdenas L, Lovy-Wheeler A, Wilsen KL, Hepler PK (2005). Actin polymerization promotes the reversal of streaming in the apex of pollen tubes. Cell Motil Cytoskeleton.

[9] Chang Z, Chen Z, Wang N, Xie G, Lu J, Yan W, Zhou J, Tang X, Deng XW (2016). Construction of a male sterility system for hybrid rice breeding and seed production using a nuclear male sterility gene. Proc Natl Acad Sci.

[10] Chen L, Liu Y-G (2014). Male sterility and fertility restoration in crops. Annu Rev Plant Biol.

[11] Conesa A, Götz S, García-Gómez JM, Terol J, Talón M, Robles MJB. Blast2GO: a universal tool for annotation, visualization and analysis in functional genomics research. 2005;21(18):3674–6. 10.1093/bioinformatics/bti610 16081474

[12] Cui L, Fan Y, Xu H, Li C, Guo Z (1999). Discovery and genetic identification of male-sterile oat in China. Zuo Wu Xue Bao.

[13] Cui X, Wang Q, Yin W, Xu H, Wilson ZA, Wei C, Pan S, Zhang D (2012). PMRD: a curated database for genes and mutants involved in plant male reproduction. BMC Plant Biol.

[14] Dixon RA, Achnine L, Kota P, Liu CJ, Reddy M, Wang L (2002). The phenylpropanoid pathway and plant defence—a genomics perspective. Mol Plant Pathol.

[15] Dorion S, Lalonde S, Saini HS (1996). Induction of male sterility in wheat by meiotic-stage water deficit is preceded by a decline in invertase activity and changes in carbohydrate metabolism in anthers. Plant Physiol.

[16] Dumas C, Knox R (1983). Callose and determination of pistil viability and incompatibility. Theor Appl Genet.

[17] Frankel R, Izhar S, Nitsan J (1969). Timing of callase activity and cytoplasmic male sterility in petunia. Biochem Genet.

[18] Franklin-Tong VE (1999). Signaling and the modulation of pollen tube growth. Plant Cell.

[19] Gibbon BC, Kovar DR, Staiger CJ (1999). Latrunculin B has different effects on pollen germination and tube growth. Plant Cell.

[20] Goetz M, Godt DE, Guivarc'h A, Kahmann U, Chriqui D, Roitsch T (2001). Induction of male sterility in plants by metabolic engineering of the carbohydrate supply. Proc Natl Acad Sci.

[21] Goldberg RB, Beals TP, Sanders PM (1993). Anther development: basic principles and practical applications. Plant Cell.

[22] Grace SC, Logan BA (2000). Energy dissipation and radical scavenging by the plant phenylpropanoid pathway. Philos Transact Royal Soc London B.

[23] Haas BJ, Alexie P, Moran Y, Manfred G, Blood PD (2013). De novo transcript sequence reconstruction from rna-seq using the trinity platform for reference generation and analysis. Nat Protoc.

[24] Heslop-Harrison J. Wall pattern formation in angiosperm microsporogenesis. Symp Soc Exp Biol. 1971. 4940549

[25] Jain S (1959). Male sterility in flowering plants. Bibliogr. Genet. 18: 101-166.. 1968. Gynodioecy in Origanum vulgare: computer simulation of a model. Nature.

[26] Jiang L, Yang S-L, Xie L-F, San Puah C, Zhang X-Q, Yang W-C, Sundaresan V, Ye D (2005). VANGUARD1 encodes a pectin methylesterase that enhances pollen tube growth in the Arabidopsis style and transmitting tract. Plant Cell.

[27] Kaul, M. L. (1988). Gramineae (grass family). Male Sterility in Higher Plants, Springer**:** 431–614.

[28] Kaul, M. L. (2012). Male sterility in higher plants, Springer Science & Business Media.

[29] Kihara, H. (1959). "Fertility and morphological variation in the substitution backcrosses of the hybrid.

[30] Kolde R (2015). Pheatmap: pretty heatmaps. Triticum vulgare× Aegilops caudata. Proc X Int Congr Genet.

[31] Langmead B, Salzberg SL (2012). Fast gapped-read alignment with bowtie 2. Nat Methods.

[32] Laser KD, Lersten NR (1972). Anatomy and cytology of microsporogenesis in cytoplasmic male sterile angiosperms. Bot Rev.

[33] Li B, Dewey CN. Rsem: accurate transcript quantification from rna-seq data with or without a reference genome. Bmc Bioinformatics. 2011. 10.1186/1471-2105-12-323 PMC3163565 21816040

[34] Li S, Yang D, Zhu Y (2007). Characterization and use of male sterility in hybrid rice breeding. J Integr Plant Biol.

[35] Longin CFH, Mühleisen J, Maurer HP, Zhang H, Gowda M, Reif JC (2012). Hybrid breeding in autogamous cereals. Theor Appl Genet.

[36] MARKOVIČ, O. and H. JÖRNVALL (1986). "Pectinesterase." Eur J Biochem 158(3): 455–462. 10.1111/j.1432-1033.1986.tb09775.x 3732279

[37] Mascarenhas JP (1975). The biochemistry of angiosperm pollen development. Bot Rev.

[38] McCormick S (2004). Control of male gametophyte development. Plant Cell.

[39] Mérai Z, Chumak N, García-Aguilar M, Hsieh T-F, Nishimura T, Schoft VK, Bindics J, Ślusarz L, Arnoux S, Opravil S (2014). The AAA-ATPase molecular chaperone Cdc48/p97 disassembles sumoylated centromeres, decondenses heterochromatin, and activates ribosomal RNA genes. Proc Natl Acad Sci.

[40] Mistry J, Finn RD, Eddy SR, Bateman A, Punta M. Challenges in homology search: HMMER3 and convergent evolution of coiled-coil regions. Nucleic Acids Res. 2013;41(12):e121–1. 10.1093/nar/gkt263 PMC3695513 23598997

[41] Mu H, Ke J, Liu W, Zhuang C, Yip W (2009). UDP-glucose pyrophosphorylase2 (OsUgp2), a pollen-preferential gene in rice, plays a critical role in starch accumulation during pollen maturation. Chin Sci Bull.

[42] Nonomura K-I, Nakano M, Fukuda T, Eiguchi M, Miyao A, Hirochika H, Kurata N (2004). The novel gene HOMOLOGOUS PAIRING ABERRATION IN RICE MEIOSIS1 of rice encodes a putative coiled-coil protein required for homologous chromosome pairing in MEIOSIS. Plant Cell.

[43] Pertea G, Huang X, Liang F, Antonescu V, Sultana R (2003). Tigr gene indices clustering tools (tgicl): a software system for fast clustering of large Est datasets. Bioinformatics.

[44] Piršelová B, Matušíková I (2013). Callose: the plant cell wall polysaccharide with multiple biological functions. Acta Physiol Plant.

[45] Quevillon E, Silventoinen V, Pillai S, Harte N, Mulder N (2005). Interproscan: protein domains identifier. Nucleic Acids Res.

[46] Riaño-Pachón DM, Ruzicic S, Dreyer I, Mueller-Roeber B (2007). Plntfdb: an integrative plant transcription factor database. Bmc Bioinform.

[47] Rice P, Longden I, Bleasby A (2000). Emboss: the european molecular biology open software suite. Trends Genet.

[48] Reich J, Brinkman M (1984). Inheritance of groat protein percentage in Avena sativa L.× A. Fatua L. crosses. Euphytica.

[49] Reznickova S, Dickinson H (1982). Ultrastructural aspects of storage lipid mobilization in the tapetum of Lilium hybrida var. enchantment. Planta.

[50] Rhee SY, Somerville CR (1998). Tetrad pollen formation in quartet mutants of Arabidopsis thaliana is associated with persistence of pectic polysaccharides of the pollen mother cell wall. Plant J.

[51] Scott RJ, Spielman M, Dickinson HG (2004). Stamen structure and function. Plant Cell.

[52] Sheoran IS, Saini HS (1996). Drought-induced male sterility in rice: changes in carbohydrate levels and enzyme activities associated with the inhibition of starch accumulation in pollen. Sex Plant Reprod.

[53] Shi Z-H, Zhang C, Xu X-F, Zhu J, Zhou Q, Ma L-J, Niu J, Yang Z-N (2015). Overexpression of AtTTP affects ARF17 expression and leads to male sterility in Arabidopsis. PLoS One.

[54] Shih-Cheng L, Loung Ping Y. Hybrid rice breeding in China. Innovative approaches to rice breeding: Selected papers form the 1979 International Rice research conference. Los Banos: International Rice Research Institute; 1980.

[55] Spencer H, Norris C, Derler J, Osis D (1991). Effect of oat bran muffins on calcium absorption and calcium, phosphorus, magnesium and zinc balance in men. J Nutr.

[56] Stevens JB, Brinkman M (1986). Performance of Avena sativa L./Avena fatua L. backcross lines. Euphytica.

[57] Suwabe K, Suzuki G, Takahashi H, Shiono K, Endo M, Yano K, Fujita M, Masuko H, Saito H, Fujioka T (2008). Separated transcriptomes of male gametophyte and tapetum in rice: validity of a laser microdissection (LM) microarray. Plant Cell Physiol.

[58] Tarazona S, Garcã A-Alcalde F, Dopazo J, Ferrer A, Conesa A (2011). Differential expression in rna-seq: a matter of depth. Genome Res.

[59] Team RC. R: A language and environment for statistical computing. 2013.

[60] Verma DPS, Hong Z (2001). Plant callose synthase complexes. Plant Mol Biol.

[61] Vidali L, Hepler P (2001). Actin and pollen tube growth. Protoplasma.

[62] Vidali L, McKenna ST, Hepler PK (2001). Actin polymerization is essential for pollen tube growth. Mol Biol Cell.

[63] Wang, Z., J. Li, S. Chen, Y. Heng, Z. Chen, J. Yang, K. Zhou, J. Pei, H. He and X. W. Deng (2017). "Poaceae-specific MS1 encodes a phospholipid-binding protein for male fertility in bread wheat." Proceedings of the National Academy of Sciences: 2017:15570. 10.1073/pnas.1715570114 PMC5703327 29109252

[64] Welch R, McVeigh A, Murphy C (1990). Hypocholesterolaemic and other responses to oat bran intake in humans. Proc Nutr Soc.

[65] Whitford R, Fleury D, Reif JC, Garcia M, Okada T, Korzun V, Langridge P (2013). Hybrid breeding in wheat: technologies to improve hybrid wheat seed production. J Exp Bot.

[66] Yamaguchi T, Hayashi T, Nakayama K, KOIKE S (2006). Expression analysis of genes for callose synthases and rho-type small GTP-binding proteins that are related to callose synthesis in rice anther. Biosci Biotechnol Biochem.

[67] Yang C, Vizcay-Barrena G, Conner K, Wilson ZA (2007). MALE STERILITY1 is required for tapetal development and pollen wall biosynthesis. Plant Cell.

